# Outcomes after TAVI in patients with atrial fibrillation and a history of recent PCI: Results from the ENVISAGE-TAVI AF trial

**DOI:** 10.1007/s00392-024-02379-5

**Published:** 2024-01-31

**Authors:** Raúl Moreno, José Souza, Rüdiger Smolnik, Luis Nombela-Franco, Nicolas M. Van Mieghem, Christian Hengstenberg, Marco Valgimigli, James Jin, Patrick Ohlmann, George Dangas, Martin Unverdorben, Helge Möllmann

**Affiliations:** 1 Interventional Cardiology, University Hospital La Paz, Paseo La Castellana, 261, 28046 Madrid, Spain; 2 Daiichi Sankyo, Inc., Munich, Germany; 3 Interventional Cardiology, Hospital Clinico San Carlos, Madrid, Spain; 4 Department of Cardiology, Erasmus University Medical Centre, Thoraxcenter, Rotterdam, the Netherlands; 5 Department of Internal Medicine II, Division of Cardiology, Vienna General Hospital, Medical University, Vienna, Austria; 6 Division of Cardiology, Cardiocentro Ticino Institute, Ente Ospedaliero Cantonale, Università Della Svizzera Italiana (USI) and University of Berne, Berne, Switzerland; 7 Daiichi Sankyo, Inc, Basking Ridge, NJ USA; 8 Division of Cardiovascular Medicine, University Hospital of Strasbourg, Strasbourg, France; 9 Mount Sinai Hospital, Zena and Michael A. Wiener Cardiovascular Institute, New York, NY USA; 10 School of Medicine, National and Kapodistrian University of Athens, Athens, Greece; 11 Department of Internal Medicine, St. Johannes Hospital, Dortmund, Germany

**Keywords:** Atrial fibrillation, Percutaneous coronary intervention, Antiplatelet therapy, Edoxaban, Transcatheter aortic valve replacement

## Abstract

**Background:**

Patients with atrial fibrillation (AF) and a recent (≤ 90 days) percutaneous coronary intervention (PCI) undergoing transcatheter aortic valve implantation (TAVI) are at high bleeding risk due to the addition of oral antiplatelet (OAP) agents on top of oral anticoagulants. Data on outcomes of these patients are needed to optimize antithrombotic treatment.

**Methods:**

This analysis compared annualized clinical event rates in patients with and without a recent PCI enrolled in ENVISAGE-TAVI AF, a prospective, randomized, open-label, adjudicator-masked trial comparing edoxaban and vitamin K antagonists in AF patients after TAVI. The primary efficacy and safety outcomes were net adverse clinical events (NACE) and major bleeding.

**Results:**

Overall, 132 (94.3%) patients with a recent PCI (*n* = 140) received OAP after TAVI, compared with 692 (55.9%) patients without a recent PCI (*n* = 1237). Among patients with a recent PCI on OAP agents, use of dual antiplatelet therapy decreased to 5.5%, and use of single antiplatelet therapy (SAPT) increased to 78.0% over 3 months post-randomization. Conversely, use of SAPT predominated at all time points in patients without a recent PCI history. There were no significant differences in the incidence of NACE or other outcomes assessed, except for major bleeding events, which were more frequent in patients with vs without a recent PCI history (hazard ratio [95% confidence interval]: 2.17 [1.27, 3.73]; *P* = 0.005).

**Conclusions:**

Patients with AF undergoing TAVI with a recent PCI have a similar risk of ischemic events and mortality, but an increased risk of major bleeding compared with patients without a recent PCI.

**Graphical abstract:**

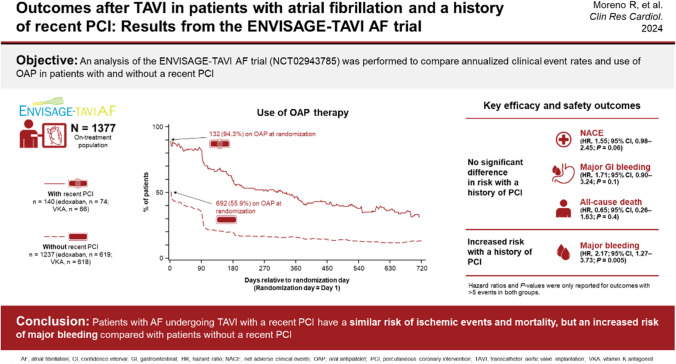

**Supplementary Information:**

The online version contains supplementary material available at 10.1007/s00392-024-02379-5.

## Background

A high proportion (~ 50%) of patients undergoing transcatheter aortic valve implantation (TAVI) have a history of coronary artery disease, and 14% to 34% had a previous percutaneous coronary intervention (PCI) [1–3]. There is no evidence supporting routine PCI in patients undergoing TAVI that present with significant coronary stenosis [4], but current guidelines recommend PCI for > 70% stenosis located in proximal segments [5]. Furthermore, there are no specific recommendations related to the timing of TAVI after PCI or the use of antiplatelet therapy in patients undergoing TAVI following recent PCI [5], but physicians may delay TAVI in patients with recent PCI [4].

Patients with atrial fibrillation (AF) and recent PCI need oral antiplatelet (OAP) therapy to prevent stent thrombosis on top of treatment with an oral anticoagulant (OAC) [5–8]. Furthermore, patients with recent PCI and coronary stent implantation undergoing TAVI may have an underlying higher risk of bleeding caused by previous major bleeding, comorbidities, adherence problems, lifestyle, or occupation [5, 8]. There is a lack of data on the use of non–vitamin K antagonist oral anticoagulant (NOAC) and clinical outcomes in patients with AF undergoing TAVI with a recent PCI; furthermore, the optimal antithrombotic therapy for these patients is not well understood. In this subanalysis from the ENVISAGE-TAVI AF trial, we assessed clinical outcomes in patients with or without a recent history of PCI.

## Methods

### Study design

The ENVISAGE-TAVI AF trial (NCT02943785) was a multinational, multicenter, prospective, randomized, open-label, adjudicator-masked trial in which patients with prevalent or incident AF were randomized to receive edoxaban or vitamin K antagonist (VKA) between 12 h and 7 days after a successful TAVI procedure [9]. The detailed design of the trial has previously been published [9]. The trial was conducted in accordance with the International Council for Harmonisation and the Declaration of Helsinki. The ethics committees and corresponding health authorities for each site approved the protocol. All patients provided written informed consent before enrollment.

### Study population

Patients 18 years or older with an indication for OAC due to prior or new-onset AF were eligible for enrollment. Key exclusion criteria included coexisting conditions that conferred a high risk of bleeding, unresolved serious periprocedural complications, and any contraindication per local label to edoxaban and VKA. Enrollment commenced in April 2017 and was completed in January 2020.

This prespecified subanalysis of ENVISAGE-TAVI AF included all randomized patients who received ≥ 1 dose of the assigned study drug. Data were collected for the duration of the treatment period and for up to 3 days after interruption or discontinuation of the study drug. Randomized patients were divided into 2 groups according to the presence or absence of a recent history of PCI. A recent PCI is defined as a procedure occurring within 90 days before TAVI and the day of randomization [8, 10–12]. Patients who underwent TAVI with PCI either prior to or during the study period could receive single antiplatelet therapy (SAPT) indefinitely. Dual antiplatelet therapy (DAPT) was only permitted after stenting for up to 3 months after PCI.

### Study endpoints

The primary efficacy outcome was the incidence of net adverse clinical events (NACE), defined as the composite of death from any cause, myocardial infarction, ischemic stroke, systemic thromboembolic event, valve thrombosis, or major bleeding (per the International Society on Thrombosis and Haemostasis [ISTH]: clinically overt bleeding associated with a reduced hemoglobin level, blood transfusion, symptomatic bleeding at a critical site, or death) [13]. The primary safety outcome was the incidence of major bleeding per ISTH criteria. Secondary outcomes included major gastrointestinal (GI) bleeding, intracranial hemorrhage, ischemic stroke, cardiovascular death, all-cause death, and clinically relevant nonmajor bleeding per ISTH criteria [9, 13].

### Statistical analysis

Baseline characteristics and clinical event rates were stratified by patients with and without a recent history of PCI. Statistical comparisons for baseline characteristics of patients with vs without a recent history of PCI were made using analysis of variance for numerical parameters and Fisher’s exact test for categorical parameters. Outcomes were reported as annualized event rates. Comparisons between patient groups and between treatment arms were reported as hazard ratios (HRs) with two-sided 95% confidence intervals (CIs) calculated using Cox proportional hazard regression models. Hazard ratios and *P*-values were only reported for outcomes with > 5 events in both groups. Interaction *P*-values were reported for outcomes with > 5 events in all subgroups.

## Results

### Patient disposition and PCI history

**Fig. 1:**
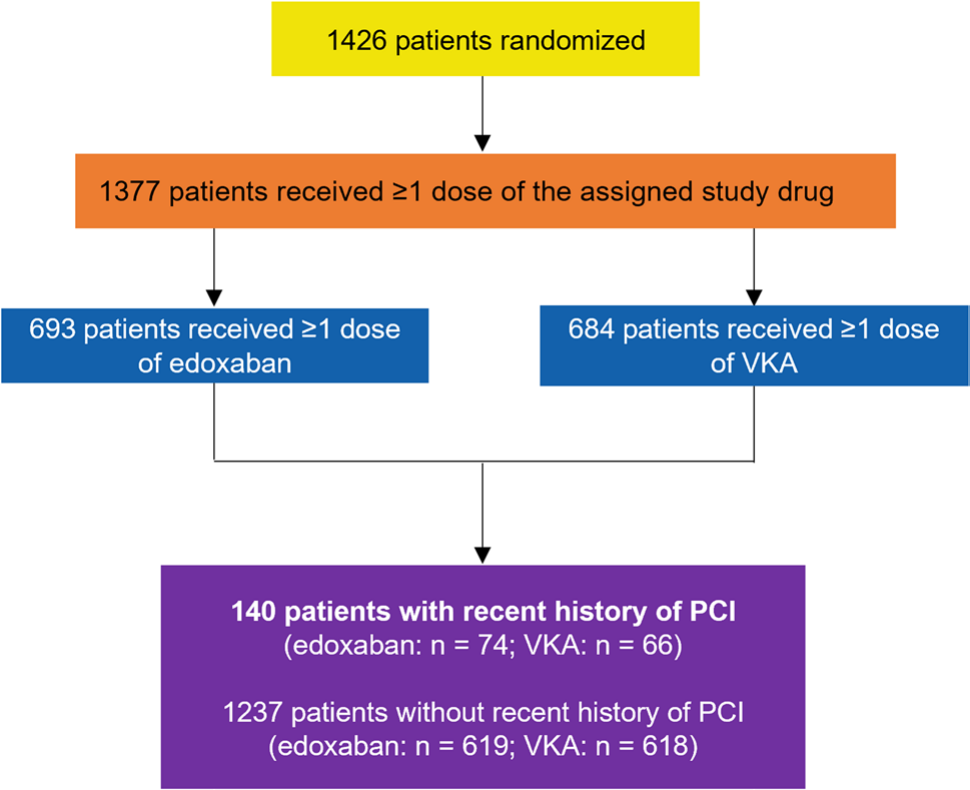
Patient disposition. *PCI* percutaneous coronary intervention; *VKA* vitamin K antagonist

**Fig. 2:**
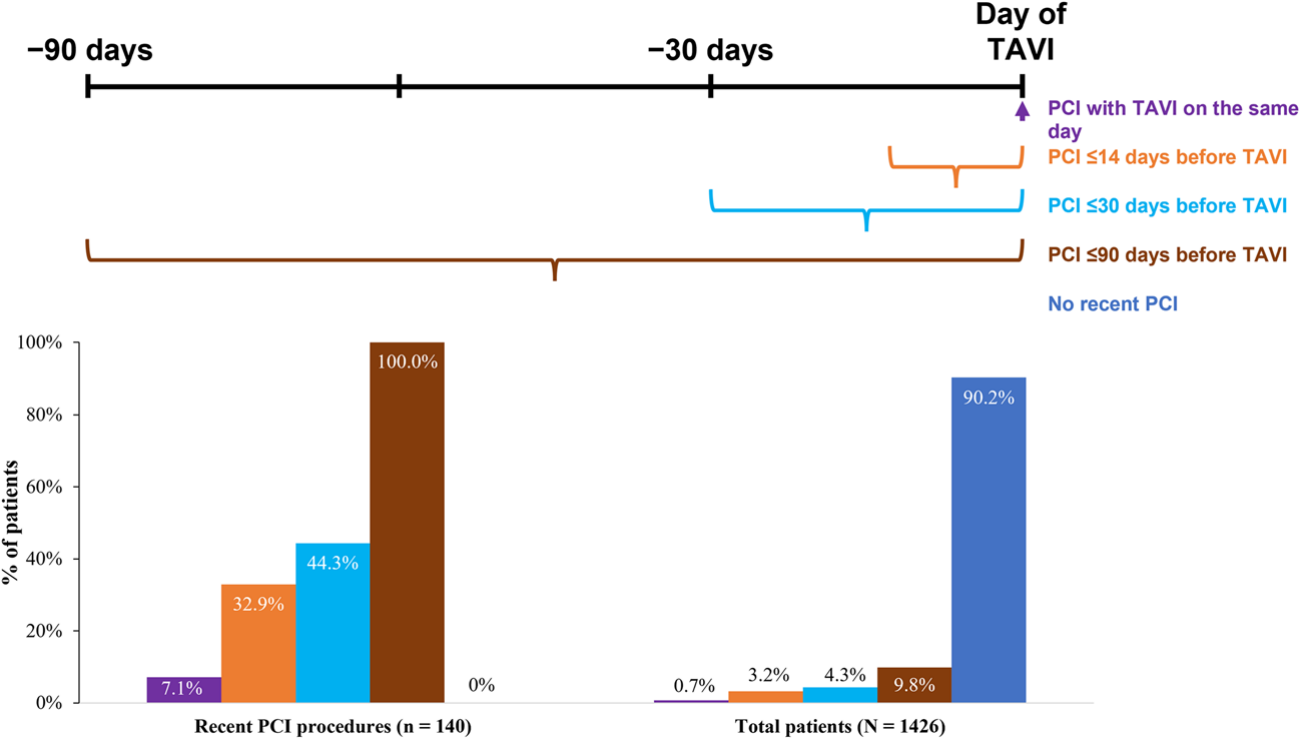
Temporal distribution of PCI. *PCI* percutaneous coronary intervention; *TAVI* transcatheter aortic valve implantation

Of the 1426 patients randomized in the ENVISAGE-TAVI AF trial, 1377 were included in this analysis (Fig. 1); 140 (9.8%) had a recent PCI, of which 62 (44.3%) were performed within 30 days before TAVI. The temporal distribution of PCI is shown in Fig. 2. Detailed PCI characteristics for patients who received a PCI within 30 days of TAVI can be found in Table S1. In the 61 patients with available data, the left anterior descending coronary artery was the most frequently treated (*n* = 32), followed by the right coronary artery (*n* = 23). Most patients (*n* = 51) received at least one drug-eluting stent.

### Patient baseline characteristics

Patients with vs without a recent PCI were more likely to have hypercholesterolemia (*P* = 0.006), prior myocardial infarction (*P* = 0.004), diabetes mellitus (*P* = 0.005), peripheral artery disease (*P* = 0.0004), and a history of stroke or transient ischemic attack (*P* = 0.001; Table 1). Baseline bleeding (HAS-BLED; hypertension, abnormal liver/renal function, stroke history, bleeding history or predisposition, labile international normalized ratio, elderly, drug/alcohol usage) and stroke (CHA_2_DS_2_-VASc; congestive heart failure, hypertension, age ≥ 75 years, diabetes mellitus, stroke or transient ischemic attack, vascular disease, age 65 to 74 years, sex category) risks were higher (*P* < 0.0001 for both) in patients with vs without a recent history of PCI. The use of a VKA pre-TAVI was more common than the use of a NOAC in patients without a PCI history (*P* < 0.0001 and *P* = 0.02). Additional baseline characteristics were similar between patients with a recent PCI history in the edoxaban and VKA arms (Table S2).

**Table 1:** Demographics and baseline characteristics in patients with or without recent PCI

Parameter	With recent PCI (*n* = 140)	Without recent PCI (*n* = 1237)	*P*-value
Age at enrollment, mean (SD)	82.1 (5.6)	82.0 (5.4)	0.8
< 65 years	2 (1.4)	8 (0.6)	
65– < 75 years	8 (5.7)	98 (7.9)	
≥ 75 years	130 (92.9)	1131 (91.4)	
Sex, male	75 (53.6)	644 (52.1)	0.8
Weight, kg, mean (SD)	75.4 (18.0)	75.3 (17.6)	0.9
Body mass index, kg/m^2^, mean (SD)	27.5 (5.3)	27.7 (5.6)	0.6
Race*			
White	110 (78.6)	1036 (83.8)	0.1
Other	30 (21.4)	201 (16.2)	
Type of AF^‡^			
Paroxysmal	70 (50.0)	499 (40.3)	**0.03**
Persistent	18 (12.9)	140 (11.3)	
Long-standing persistent	7 (5.0)	103 (8.3)	
Permanent	44 (31.4)	474 (38.3)	
With atrial flutter	1 (0.7)	17 (1.4)	
Stroke/TIA	38 (27.1)	195 (15.8)	**0.001**
Hypertension	129 (92.1)	1129 (91.3)	0.9
Coronary artery disease	101 (72.1)	640 (51.7)	** < 0.0001**
Hypercholesterolemia	112 (80.0)	852 (68.9)	**0.006**
Diabetes mellitus	67 (47.9)	439 (35.5)	**0.005**
Hospitalization for bleeding	6 (4.3)	54 (4.4)	1
Valvular heart disease	140 (100)	1237 (100)	
Non-CNS systemic thromboembolic event	10 (7.1)	60 (4.9)	0.2
Peripheral artery disease	30 (21.4)	127 (10.3)	**0.0004**
Carotid artery disease	14 (10.0)	82 (6.6)	0.2
COPD	14 (10.0)	185 (15.0)	0.1
Myocardial infarction	31 (22.1)	160 (12.9)	**0.004**
Prior major bleeding or predisposition to bleeding	12 (8.6)	107 (8.6)	1
CABG	15 (10.7)	109 (8.8)	0.4
PCI performed within 30 days before TAVI	62 (44.3)	0	** < 0.0001**
Ejection fraction, mean (SD)	54.9 (12.1)	55.6 (11.3)	0.5
CrCl, mL/min, mean (SD)^†^	56.8 (22.4)	58.4 (24.3)	0.4
CrCl ≤ 50	59 (42.1)	511 (41.3)	0.9
OAP prior to randomization	114 (81.4)	508 (41.1)	** < 0.0001**
HAS-BLED score, mean (SD)	1.9 (0.9)	1.5 (0.7)	** < 0.0001**
CHA_2_DS_2_-VASc score, mean (SD)	5.0 (1.6)	4.4 (1.3)	** < 0.0001**
Gastrointestinal disorder	55 (39.3)	443 (35.8)	0.5
Previous PPI use	57 (40.7)	543 (43.9)	0.5
Pre-TAVI use of VKA	37 (26.4)	596 (48.2)	** < 0.0001**
Pre-TAVI use of NOAC	51 (36.4)	333 (26.9)	**0.02**
No pre-TAVI use of VKA or NOAC	52 (37.1)	308 (24.9)	**0.003**
Labile INR	7 (5.0)	101 (8.2)	0.2
Indication for dose adjustment^§^	71 (50.7)	566 (45.8)	0.3
STS score, mean (SD)	6.0 (4.2)	4.8 (3.8)	**0.0005**
EuroSCORE I, mean (SD)	12.8 (9.6)	12.9 (9.9)	0.9
EuroSCORE II, mean (SD)	4.6 (3.6)	4.6 (5.7)	0.9
Edoxaban arm	74 (52.9)	619 (50.0)	0.5
VKA arm	66 (47.1)	618 (50.0)	0.5
Intracranial hemorrhage	2 (1.4)	15 (1.2)	0.7
Cigarette use (current or former)	54 (38.6)	391 (31.6)	0.1
Abnormal renal function	4 (2.9)	22 (1.8)	0.3
Abnormal liver function	0	3 (0.2)	1
Major bleed, anemia	12 (8.6)	107 (8.6)	1
Chronic drug usage**	52 (37.1)	175 (14.1)	** < 0.0001**
Excessive alcohol use	2 (1.4)	26 (2.1)	1
Hemoglobin, g/L, mean (SD)	113.3 (99.3)	115.9 (75.0)	0.7
Platelets, 10^9^/L, mean (SD)	170.9 (58.1)	157.2 (54.8)	**0.006**

Data presented as n (%) unless otherwise noted

Bold value indicate*P*< 0.05

^*^Race was reported by the investigator from information obtained from patient history. “Other” includes patients of another race and those who chose not to report race. ^†^Cockcroft–Gault formula. ^‡^Persistent defined as irregular rhythm occurring from 8 to 364 days; long-standing persistent for > 1 year. ^§^Indications for adjustment of the edoxaban dose included CrCl ≤ 50 mL/min, bodyweight of ≤ 60 kg (not used as an indication in US patients), and concomitant therapy with a P-glycoprotein inhibitor (not used as an indication in US patients). **Chronic drug usage is one component of the HAS-BLED score, including antiplatelet agents, NSAIDs

*AF* atrial fibrillation; *CABG* coronary artery bypass graft surgery; *CHA*_*2*_*DS*_*2*_*-VASc* congestive heart failure, hypertension, age ≥ 75 years, diabetes mellitus, stroke or TIA, vascular disease, age 65 to 74 years, sex category; *CNS* central nervous system; *COPD* chronic obstructive pulmonary disease; *CrCl* creatinine clearance; *HAS-BLED* hypertension, abnormal liver/renal function, stroke history, bleeding history or predisposition, labile INR, elderly, drug/alcohol usage; *INR* international normalized ratio; *NOAC* non–vitamin K antagonist oral anticoagulant; *NSAID* nonsteroidal anti-inflammatory drug; *OAP* oral antiplatelet; *PCI* percutaneous coronary intervention; *PPI* proton pump inhibitor; *SD* standard deviation; *STS* Society of Thoracic Surgeons; *TAVI* transcatheter aortic valve implantation; *TIA* transient ischemic attack; *VKA* vitamin K antagonist

### Use of oral anticoagulant and antiplatelet therapies over time

**Fig. 3:**
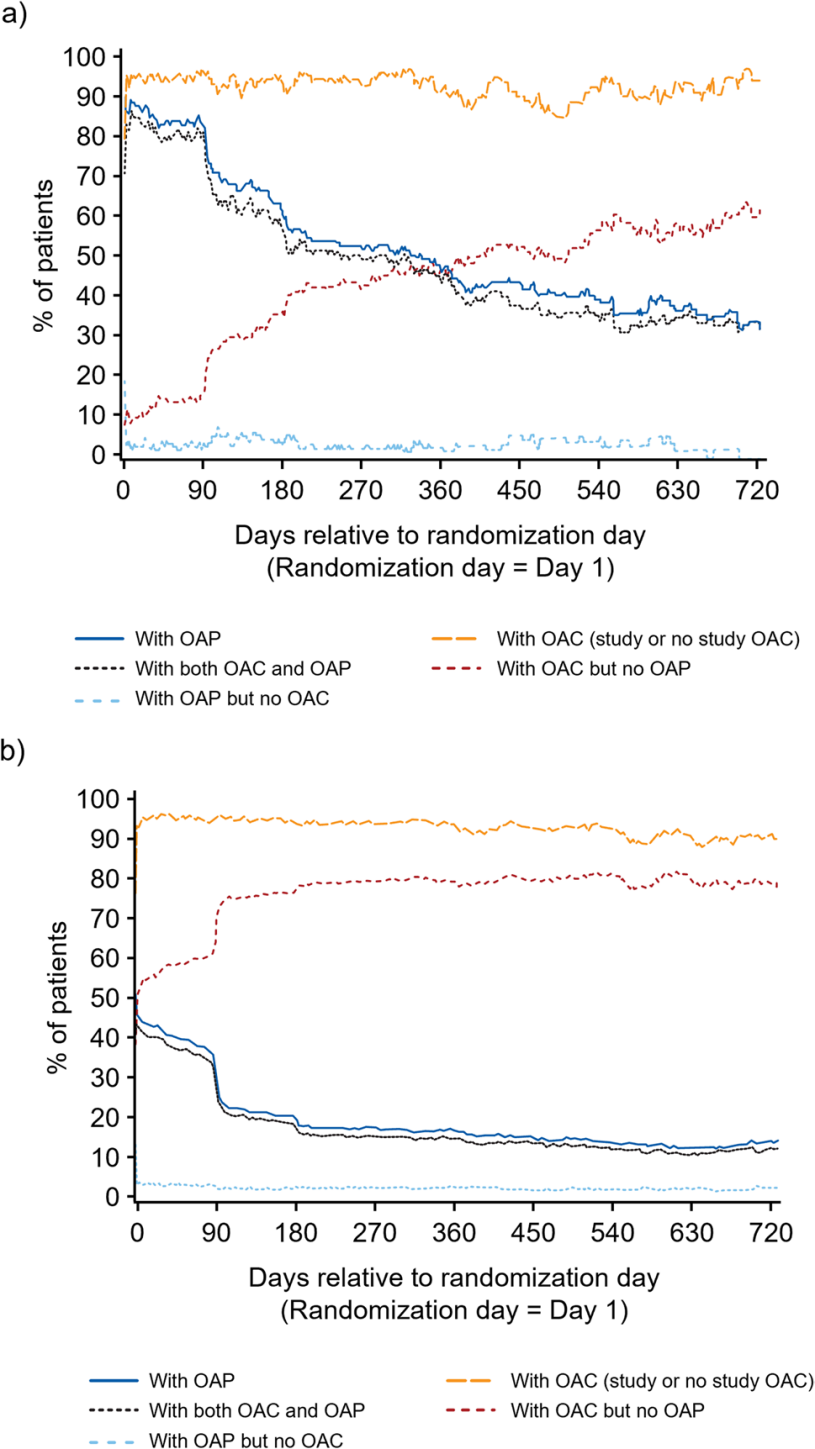
Use of OAP over time in patients with (**a**) and without (**b**) recent PCI history. *OAC* oral anticoagulation; *OAP* oral antiplatelet; *PCI* percutaneous coronary intervention

Most patients with a recent history of PCI (132 [94.3%]) received OAP therapy after TAVI (Fig. 3). After 90 days post-randomization, use of OAP decreased and continued to decline, with the majority receiving OAC without concomitant OAP at 1 year post-randomization. The use of OAP was less frequent in patients without a recent history of PCI, with 692 (55.9%) patients on OAP therapy after TAVI. This rate decreased to approximately 20% of patients around 90 days post-randomization. During the remainder of the observation period, approximately 15% of patients received concomitant OAP.

The use of SAPT and DAPT over time is shown in Fig. S1. DAPT was frequently prescribed in patients with recent PCI. Among patients with a recent history of PCI receiving OAP, the percentage of patients on SAPT (53.6%) increased over the first 3 months post-randomization, while the percentage on DAPT (37.1%) decreased. At 3 months, 78.0% of patients with a recent history of PCI received SAPT, and 5.5% received DAPT. Beyond 3 months, the use of any OAP in this subgroup was primarily SAPT.

### Recent PCI and clinical outcomes

**Fig. 4:**
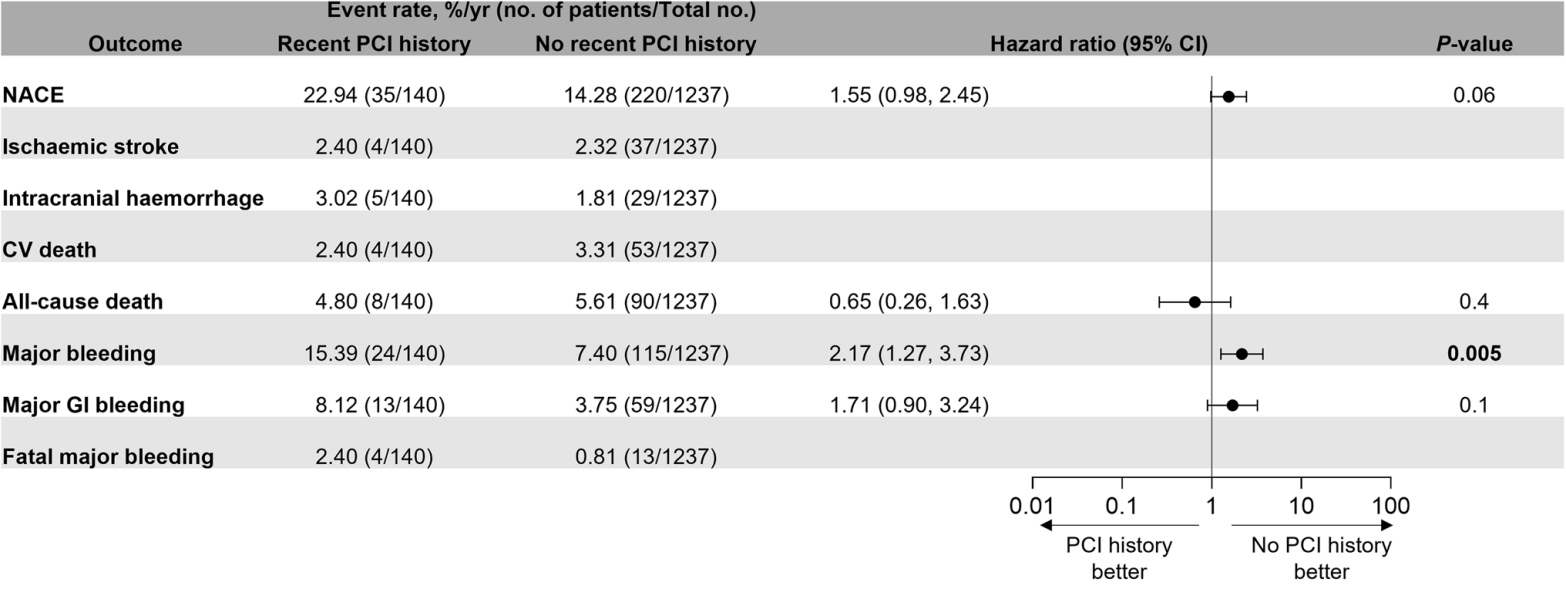
Primary efficacy and primary safety outcomes in patients with and without a recent PCI history. Outcomes are reported for the on-treatment population (ie, patients on study medication or within 3 days of previous study medication). Hazard ratios and *P*-values were only reported for outcomes with > 5 events in both groups. Bold values indicate a *P*<0.05 *CI* confidence interval; *PCI* percutaneous coronary intervention

Patients with a recent PCI had a numerically higher annualized incidence of NACE than those without a recent history of PCI (22.94%/yr vs 14.28%/yr; HR [95% CI]: 1.55 [0.98, 2.45]; *P* = 0.06; Fig. 4). This finding was attributable to patients with a recent PCI having a significantly higher annualized incidence of major bleeding events (15.39%/yr vs 7.40%/yr; HR [95% CI]: 2.17 [1.27, 3.73]; *P* = 0.005) and numerically more frequent major GI bleeding (8.12%/yr vs 3.75%/yr; HR [95% CI]: 1.71 [0.90, 3.24]; *P* = 0.1) and intracranial hemorrhage events (3.02%/yr vs 1.81%/yr) compared with those without a recent history of PCI. Differences between patients with and without a recent history of PCI in the time to first NACE and first major bleeding event were evident around 90 and 45 days post-randomization, respectively (Fig. S2). The risk of ischemic stroke was similar in patients with and without a recent PCI (2.40%/yr vs 2.32%/yr). Annualized incidences of cardiovascular death were 2.40%/yr vs 3.31%/yr and all-cause death were 4.80%/yr vs 5.61%/yr (HR [95% CI]: 0.65 [0.26, 1.63]; *P* = 0.4) in patients with vs those without a recent PCI (Fig. 4, Fig. S2).

**Fig. 5:**
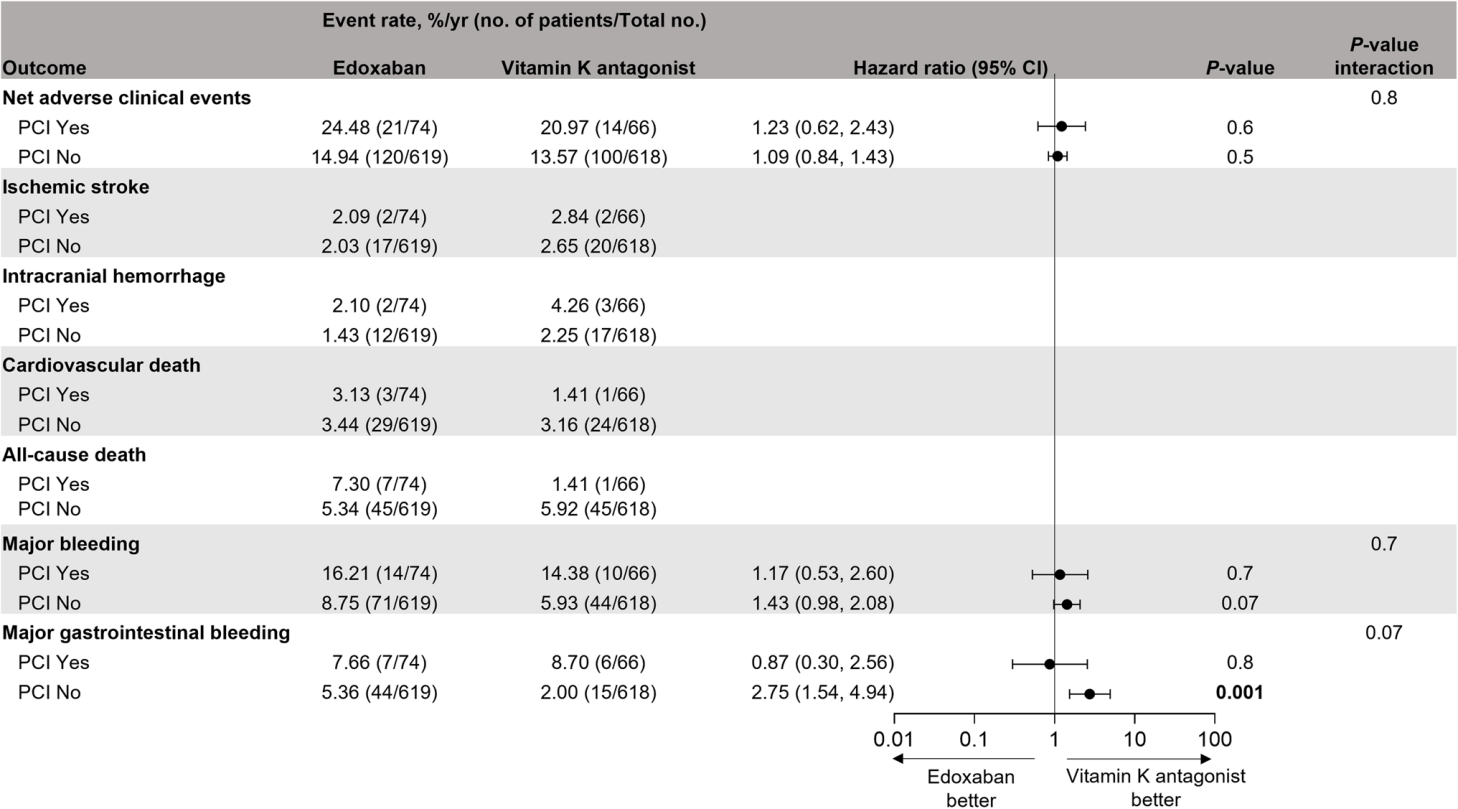
Clinical outcomes in patients with and without recent PCI receiving edoxaban or VKA. Outcomes are reported for the on-treatment population (ie, patients on study medication or within 3 days of previous study medication). Hazard ratios and *P*-values were only reported for outcomes with > 5 events in both treatment groups. Interaction *P*-values were provided for outcomes with > 5 events in all subgroups. Bold values indicate a *P*<0.05 *CI* confidence interval; *PCI* percutaneous coronary intervention

Regardless of a recent history of PCI, the incidences of NACE and major bleeding were similar between patients receiving edoxaban vs those receiving VKA (Fig. 5). In patients without a recent PCI, the incidence of major GI bleeding was significantly higher for patients receiving edoxaban compared with those receiving VKA (5.36%/yr vs 2.00%/yr; HR [95% CI]: 2.75 [1.54, 4.94]; *P* = 0.001). For patients with a recent PCI, of the first 24 major bleeding events, 12 were GI (Table S3), but the annualized event rate of major GI bleeding was similar between treatment arms (7.66%/yr vs 8.70%/yr; HR [95% CI]: 0.87 [0.30, 2.56]; *P* = 0.8). There was no significant interaction between the patient group and the treatment group for any of the outcomes.

## Discussion

In this subanalysis of patients from the ENVISAGE-TAVI AF trial with a recent history of PCI after undergoing TAVI, the incidence of NACE and the risk of major bleeding and major GI bleeding were similar in patients receiving edoxaban and VKA. This finding was not shown with any other NOAC, confirming that edoxaban is a worthy alternative to VKA in this special population.

In this study, 9.8% of patients with AF undergoing TAVI had a recent PCI. In comparison, this is lower than in a study that found approximately 18% of patients with AF undergoing TAVI had a recent PCI [14]. Furthermore, these results were also lower in patients with aortic stenosis undergoing TAVI, in which 14% to 34% have a history of PCI [2, 3]. This relatively low prevalence of a previous PCI in the ENVISAGE-TAVI AF trial is likely attributable to the focus on patients who had undergone a recent PCI (≤ 90 days), whereas the previous studies had a 1- to 2-year follow-up period [2, 3, 8, 14].

Another study finding was that patients with a recent history of PCI had a higher atherosclerotic risk profile (higher prevalence of previous cardiovascular ischemic events and risk factors) and higher HAS-BLED and CHA_2_DS_2_-VASc risk scores than those without a recent PCI, as may be expected since PCI is performed in patients with lesions with high stenoses and/or patients with angina. This clinical profile carried neither a higher risk of stroke nor all-cause or cardiovascular death, but bleeding events occurred more frequently in this subgroup. Previous research in anticoagulated patients with AF showed both the HAS-BLED and CHA_2_DS_2_-VASc risk scores were moderate predictors of stroke, major bleeding, and mortality events [15]. These 2 scores performed similarly in the prediction of major bleeding events, suggesting that patients with an increased risk of stroke also have an increased bleeding risk [15, 16]. However, these previous analyses were not specific to patients with AF undergoing TAVI in contrast to the current study.

Data from previous studies involving patients with AF undergoing TAVI show that simplifying antithrombotic treatment offers a better safety profile (i.e., reduced bleeding complications) without increasing the risk of thromboembolic events [17, 18]. Thus, the consensus is to use OAC alone in patients with AF undergoing TAVI unless there is an established indication for antiplatelet therapy [8]. The presence of a recent PCI in patients with AF undergoing TAVI has a clear implication for antithrombotic therapy. Still, the need for coadministration of antiplatelet drugs and OAC increases the risk of bleeding events. Previous studies in patients with a recent PCI have investigated DAPT de-escalation strategies and demonstrated a reduction in bleeding events without an increase in ischemic events [19, 20]. The study results presented here showed that patients with a recent history of PCI received OAP approximately twice as frequently as patients without a recent PCI and had an increased rate of major bleeding. Further research exploring de-escalation of OAP in patients with AF undergoing TAVI with a recent PCI may be warranted.

Some practical implications of our findings may be related to the procedural aspects of PCI in patients with AF undergoing TAVI. First, if the clinical situation allows, it may be convenient to delay TAVI in patients having undergone PCI until antithrombotic treatment can be simplified. Although there are no specific recommendations about timing in patients with AF needing TAVI after a recent PCI [5], it is not uncommon for physicians to delay TAVI in these patients [4]. In the ACTIVATION study, patients were randomly assigned to receive PCI prior to TAVI. The median time from randomization to TAVI was 41 days in the PCI group and 27 days in the no-PCI group [4]. Second, the use of drug-eluting balloons instead of coronary stents may reduce the need for antiplatelet therapy and may be an option for patients at high bleeding risk, including those undergoing TAVI [21, 22]. Third, drug-eluting stents with proven efficacy and safety under short-term (1 month) DAPT may provide clinical benefits in patients with AF after TAVI [23]. Clinicians may consider simplifying and shortening antiplatelet therapy according to ischemic and bleeding risk in these patients.

The ENVISAGE-TAVI AF trial demonstrated that edoxaban was noninferior to VKA in patients with AF undergoing TAVI with regards to the primary efficacy endpoint of NACE [9]. However, more patients on edoxaban vs VKA had increased major bleeding and major GI bleeding events [9]. Here, in patients with a recent PCI, the incidences of NACE, major bleeding, and major GI bleeding were similar between treatment arms, in this population at high risk of ischemic events and cardiovascular mortality. In patients without a recent history of PCI, there was no difference between treatment arms in the incidence of NACE. However, the incidence of major GI bleeding was higher for edoxaban vs VKA, and subsequently, the frequency of major bleeding events was numerically higher for edoxaban vs VKA. Notably, this trend was not observed in patients with a recent history of PCI. This could suggest that edoxaban may not increase the risk of major bleeding relative to VKA in instances where use of OAP is appropriately indicated.

In the ENTRUST-AF PCI (Edoxaban-based vs Vitamin K Antagonist-based Antithrombotic Regimen After Successful Coronary Stenting in Patients with Atrial Fibrillation) trial, 1506 patients with AF having undergone a successful PCI were randomized to either edoxaban plus a P2Y12 inhibitor for 12 months or to a VKA in combination with a P2Y12 inhibitor and aspirin for 1–12 months [24]. The patients in ENTRUST were younger with a lower cardiovascular risk compared to patients in the ENVISAGE-TAVI AF study. The main efficacy outcome (combination of cardiovascular death, stroke, systemic embolic events, myocardial infarction, and definitive stent thrombosis) was similar in both groups of patients in ENTRUST. The annualized rate of major bleeding was lower in patients allocated to edoxaban (20.7%) vs VKA (25.6%), although superiority was not met (*P* = 0.001 for noninferiority, *P* = 0.1154 for superiority). The present subanalysis from the ENVISAGE-TAVI AF trial extends the finding that edoxaban is noninferior to VKA in patients with AF with recent PCI to the complex group of patients with AF who have undergone TAVI.

### Limitations

There are several limitations to this subanalysis, including the small number of patients with a recent history of PCI. Additionally, details on PCI procedures were only available for 61 out of 140 patients. Although information on the number of stents implanted was not specifically collected, the numbers of index lesion stents add up to 61, which is also the total number of PCI procedures for which any details are available, suggesting it is unlikely that more than one stent was used for PCI of the index lesion. There were no specific recommendations included in the ENVISAGE-TAVI AF trial regarding the PCI indication and the timing from PCI to TAVI. Notably, patients undergoing TAVI who also had concomitant coronary artery disease or recently underwent PCI were eligible for enrollment in the trial, reflecting the real-world population of patients undergoing TAVI. Additionally, when the ENVISAGE-TAVI AF trial was performed, data from randomized trials providing insight into simpler antithrombotic regimens after TAVI were not available. The rate of bleeding events in patients with AF undergoing TAVI treated with the simpler antithrombotic regimens currently recommended may be lower than what was observed in the trial. It is important to note that the results of this study are only applicable to patients undergoing TAVI who are receiving OAC due to AF, not to the general population of patients undergoing TAVI. Furthermore, the study may not have enrolled patients with clinical events, such as intracranial hemorrhage occurring shortly after PCI. This possibility may have introduced selection bias through the exclusion of patients with a recent history of PCI who experienced a bleeding or ischemic event after PCI. However, this potential limitation would have impacted the edoxaban and VKA arms equally. Larger studies adequately powered to statistically compare outcomes with edoxaban to VKA in patients with and without a recent history of PCI are needed to confirm the results presented here.

## Conclusions

Among patients with prior and new-onset AF undergoing TAVI, those with vs without a recent history of PCI had a similar risk of ischemic events and cardiovascular mortality and a significantly higher risk of major bleeding events. Additionally, patients with a recent PCI who were treated with edoxaban had similar incidences of NACE, major bleeding, and major GI bleeding events compared with those treated with VKA. These results indicate that edoxaban is an alternative to VKA in this very high-risk patient population.

## Supplementary Information

Below is the link to the electronic supplementary material.

Supplementary file1 (DOCX 157 KB)

## Data Availability

The data underlying this article will be shared on reasonable request to the corresponding author.

## References

[1] Faroux L, Guimaraes L, Wintzer-Wehekind J et al (2019) Coronary artery disease and transcatheter aortic valve replacement: JACC state-of-the-art review. J Am Coll Cardiol 74(3):362–372 31319919 10.1016/j.jacc.2019.06.012

[2] Popma JJ, Deeb GM, Yakubov SJ et al (2019) Transcatheter aortic-valve replacement with a self-expanding valve in low-risk patients. N Engl J Med 380(18):1706–1715 30883053 10.1056/NEJMoa1816885

[3] Smith CR, Leon MB, Mack MJ et al (2011) Transcatheter versus surgical aortic-valve replacement in high-risk patients. N Engl J Med 364(23):2187–2198 21639811 10.1056/NEJMoa1103510

[4] Patterson T, Clayton T, Dodd M et al (2021) ACTIVATION (PercutAneous coronary inTervention prIor to transcatheter aortic VAlve implantaTION): A randomized clinical trial. JACC Cardiovasc Interv 14(18):1965–1974 34556269 10.1016/j.jcin.2021.06.041

[5] Vahanian A, Beyersdorf F, Praz F et al (2022) 2021 ESC/EACTS guidelines for the management of valvular heart disease: Developed by the task force for the management of valvular heart disease of the European Society of Cardiology (ESC) and the European Association for Cardio-Thoracic Surgery (EACTS). Rev Esp Cardiol (Engl Ed) 75(6):524 35636831 10.1016/j.rec.2022.05.006

[6] Neumann FJ, Sousa-Uva M, Ahlsson A et al (2019) 2018 ESC/EACTS guidelines on myocardial revascularization. Eur Heart J 40(2):87–165 30615155 10.1093/eurheartj/ehy855

[7] Angiolillo DJ, Goodman SG, Bhatt DL et al (2016) Antithrombotic therapy in patients with atrial fibrillation undergoing percutaneous coronary intervention: A North American perspective-2016 update. Circ Cardiovasc Interv 9(11):e004395 27803042 10.1161/CIRCINTERVENTIONS.116.004395

[8] Ten Berg J, Sibbing D, Rocca B et al (2021) Management of antithrombotic therapy in patients undergoing transcatheter aortic valve implantation: A consensus document of the ESC working group on thrombosis and the European Association of Percutaneous Cardiovascular Interventions (EAPCI), in collaboration with the ESC Council on valvular heart disease. Eur Heart J 42(23):2265–2269 33822924 10.1093/eurheartj/ehab196

[9] Van Mieghem NM, Unverdorben M, Hengstenberg C et al (2021) Edoxaban versus vitamin K antagonist for atrial fibrillation after TAVR. N Engl J Med 385:2150–2610 34449183 10.1056/NEJMoa2111016

[10] Baron TH, Kamath PS, McBane RD (2013) Management of antithrombotic therapy in patients undergoing invasive procedures. N Engl J Med 368(22):2113–2124 23718166 10.1056/NEJMra1206531

[11] Hindricks G, Potpara T, Dagres N et al (2021) 2020 ESC guidelines for the diagnosis and management of atrial fibrillation developed in collaboration with the European Association for Cardio-Thoracic Surgery (EACTS): The task force for the diagnosis and management of atrial fibrillation of the European Society of Cardiology (ESC) developed with the special contribution of the European heart rhythm association (EHRA) of the ESC. Eur Heart J 42(5):373–498 32860505 10.1093/eurheartj/ehaa612

[12] January CT, Wann LS, Calkins H et al (2019) AHA/ACC/HRS focused update of the 2014 AHA/ACC/HRS guideline for the management of patients with atrial fibrillation: A report of the American College of Cardiology/American Heart Association task force on clinical practice guidelines and the Heart Rhythm Society. J Am Coll Cardiol 74(1):104–132 10.1016/j.jacc.2019.01.011 30703431

[13] Schulman S, Kearon C (2005) Definition of major bleeding in clinical investigations of antihemostatic medicinal products in non-surgical patients. J Thromb Haemost 3(4):692–694 15842354 10.1111/j.1538-7836.2005.01204.x

[14] Stortecky S, Buellesfeld L, Wenaweser P et al (2013) Atrial fibrillation and aortic stenosis: Impact on clinical outcomes among patients undergoing transcatheter aortic valve implantation. Circ Cardiovasc Interv 6(1):77–84 23386662 10.1161/CIRCINTERVENTIONS.112.000124

[15] Morrone D, Kroep S, Ricci F et al (2020) Mortality prediction of the CHA(2)DS(2)-VASc score, the HAS-BLED score, and their combination in anticoagulated patients with atrial fibrillation. J Clin Med 9(12):3987 33317069 10.3390/jcm9123987 PMC7764787

[16] Yao X, Gersh BJ, Sangaralingham LR et al (2017) Comparison of the CHA(2)DS(2)-VASc, CHADS(2), HAS-BLED, ORBIT, and ATRIA risk scores in predicting non-vitamin K antagonist oral anticoagulants-associated bleeding in patients with atrial fibrillation. Am J Cardiol 120(9):1549–1556 28844514 10.1016/j.amjcard.2017.07.051

[17] Nijenhuis VJ, Brouwer J, Delewi R et al (2020) Anticoagulation with or without clopidogrel after transcatheter aortic-valve implantation. N Engl J Med 382(18):1696–1707 32223116 10.1056/NEJMoa1915152

[18] Sherwood MW, Gupta A, Vemulapalli S et al (2021) Variation in antithrombotic therapy and clinical outcomes in patients with preexisting atrial fibrillation undergoing transcatheter aortic valve replacement: Insights from the Society of Thoracic Surgeons/American College of Cardiology Transcatheter Valve Therapy Registry. Circ Cardiovasc Interv 14(4):e009963 33877866 10.1161/CIRCINTERVENTIONS.120.009963 PMC8300574

[19] Cuisset T, Deharo P, Quilici J et al (2017) Benefit of switching dual antiplatelet therapy after acute coronary syndrome: The TOPIC (timing of platelet inhibition after acute coronary syndrome) randomized study. Eur Heart J 38(41):3070–3078 28510646 10.1093/eurheartj/ehx175

[20] Kim HS, Kang J, Hwang D et al (2020) Prasugrel-based de-escalation of dual antiplatelet therapy after percutaneous coronary intervention in patients with acute coronary syndrome (HOST-REDUCE-POLYTECH-ACS): An open-label, multicentre, non-inferiority randomised trial. Lancet 396(10257):1079–1089 32882163 10.1016/S0140-6736(20)31791-8

[21] Rissanen TT, Uskela S, Eranen J et al (2019) Drug-coated balloon for treatment of de-novo coronary artery lesions in patients with high bleeding risk (DEBUT): A single-blind, randomised, non-inferiority trial. Lancet 394(10194):230–239 31204115 10.1016/S0140-6736(19)31126-2

[22] Urban P, Meredith IT, Abizaid A et al (2015) Polymer-free drug-coated coronary stents in patients at high bleeding risk. N Engl J Med 373(21):2038–2047 26466021 10.1056/NEJMoa1503943

[23] Windecker S, Latib A, Kedhi E et al (2020) Polymer-based or polymer-free stents in patients at high bleeding risk. N Engl J Med 382(13):1208–1218 32050061 10.1056/NEJMoa1910021

[24] Vranckx P, Valgimigli M, Eckardt L et al (2019) Edoxaban-based versus vitamin K antagonist-based antithrombotic regimen after successful coronary stenting in patients with atrial fibrillation (ENTRUST-AF PCI): A randomised, open-label, phase 3b trial. Lancet 394(10206):1335–1343 31492505 10.1016/S0140-6736(19)31872-0

