# Supplementary material for: Outcomes after TAVI in patients with atrial fibrillation and a history of recent PCI: Results from the ENVISAGE-TAVI AF trial
Source: Clin Res Cardiol. 2024 Jan 31;114(3):313–22. doi: 10.1007/s00392-024-02379-5 (PMC11914355; doi:10.1007/s00392-024-02379-5)
Supplement: Supplementary file 1 — Supplementary file1 (DOCX 157 KB) [file 392_2024_2379_MOESM1_ESM.docx]

**Supplemental material**

**Table S1** Detailed PCI characteristics

| **Parameter** | **N = 61**^a^ |
| --- | --- |
| **Segment** |  |
| Left main coronary artery | 3 |
| Left anterior descending artery | 32 |
| Circumflex coronary artery | 15 |
| Right coronary artery | 23 |
| **Index lesion stent type** |  |
| Drug-eluting stent | 51 |
| Bare-metal stent | 2 |
| None/other^b^ | 8 |
| **Type of lesion** |  |
| De novo | 53 |
| In-stent restenosis | 5 |
| **Mode of stenting**^c^ |  |
| Direct stenting | 15 |
| Predilatation | 41 |
| **Vascular access site** |  |
| Femoral | 22 |
| Radial/brachial | 28 |
| Radial + femoral | 1 |
| Not available | 10 |

Data presented as number of patients. ^a^Patients with missing data in the eCRF are not included. ^b^Includes use of drug-eluting balloons. ^c^Missing data for specific parameters not listed separately.

*eCRF* electronic case report form; *PCI* percutaneous coronary intervention.

**Table S2** Demographics and baseline characteristics by treatment arm in patients with a recent PCI history

| **Parameter** | **Edoxaban**  **(n = 74)** | **VKA**  **(n = 66)** | ***P*-value** |
| --- | --- | --- | --- |
| **Age at enrollment, years, mean (SD)** | 82.3 (5.6) | 82.0 (5.7) | 0.8 |
| <65 years | 1 (1.4) | 1 (1.5) |  |
| 65–<75 years | 5 (6.8) | 3 (4.5) |  |
| ≥75 years | 68 (91.9) | 62 (93.9) |  |
| **Male** | 42 (56.8) | 33 (50.0) | 0.5 |
| **Weight, kg, mean (SD)** | 74.5 (16.9) | 76.4 (19.4) | 0.5 |
| **Body mass index, kg/m^2^, mean (SD)** | 27.2 (5.3) | 27.7 (5.2) | 0.6 |
| **Race*** |  |  |  |
| White | 56 (75.7) | 54 (81.8) | 0.4 |
| Other | 18 (24.3) | 12 (18.2) |  |
| **Type of AF**^‡^ |  |  |  |
| Paroxysmal | 37 (50.0) | 33 (50.0) | 1 |
| Persistent | 8 (10.8) | 10 (15.2) |  |
| Long-standing persistent | 5 (6.8) | 2 (3.0) |  |
| Permanent | 23 (31.1) | 21 (31.8) |  |
| With atrial flutter | 1 (1.4) | 0 |  |
| **Valvular heart disease** | 74 (100) | 66 (100) |  |
| **Hypertension** | 66 (89.2) | 63 (95.5) | 0.2 |
| **Hypercholesterolemia** | 62 (83.8) | 50 (75.8) | 0.3 |
| **Coronary artery disease** | 57 (77.0) | 44 (66.7) | 0.2 |
| **Diabetes mellitus** | 38 (51.4) | 29 (43.9) | 0.4 |
| **Stroke/TIA** | 23 (31.1) | 15 (22.7) | 0.3 |
| **Myocardial infarction** | 17 (23.0) | 14 (21.2) | 0.8 |
| **Peripheral artery disease** | 14 (18.9) | 16 (24.2) | 0.5 |
| **COPD** | 7 (9.5) | 7 (10.6) | 1 |
| **Carotid artery disease** | 6 (8.1) | 8 (12.1) | 0.6 |
| **Ejection fraction, mean (SD)** | 54.8 (13.2) | 54.9 (10.9) | 1.0 |
| **HAS-BLED score, mean (SD)** | 2.0 (0.9) | 1.7 (0.9) | 0.1 |
| **CHA_2_DS_2_-VASc score, mean (SD)** | 5.0 (1.6) | 4.9 (1.5) | 0.7 |
| **OAP prior to randomization** | 64 (86.5) | 50 (75.8) | 0.1 |
| **Indication for dose adjustment**^§^ | 36 (48.6) | 35 (53.0) | 0.6 |
| **CrCl, mL/min, mean (SD)**^†^ | 56.7 (23.6) | 56.8 (21.2) | 1.0 |
| CrCl ≤50 | 31 (41.9) | 28 (42.4) | 1 |
| **PCI performed within 30 days before TAVI** | 34 (45.9) | 28 (42.4) | 0.7 |
| **Gastrointestinal disorder** | 32 (43.2) | 23 (34.8) | 0.4 |
| **Previous PPI use** | 32 (43.2) | 25 (37.9) | 0.6 |
| **Chronic drug usage**** | 31 (41.9) | 21 (31.8) | 0.2 |
| **Cigarette use (current or former)** | 29 (39.2) | 25 (37.9) | 1 |
| **Pre-TAVI use of NOAC** | 29 (39.2) | 22 (33.3) | 0.5 |
| **No pre-TAVI use of VKA or NOAC** | 28 (37.8) | 24 (36.4) | 0.9 |
| **Pre-TAVI use of VKA** | 17 (23.0) | 20 (30.3) | 0.3 |
| **Prior major bleeding or predisposition to bleeding** | 8 (10.8) | 4 (6.1) | 0.4 |
| **Major bleed, anemia** | 8 (10.8) | 4 (6.1) | 0.4 |
| **CABG** | 7 (9.5) | 8 (12.1) | 0.8 |
| **Non-CNS systemic thromboembolic event** | 5 (6.8) | 5 (7.6) | 1 |
| **Labile INR** | 4 (5.4) | 3 (4.5) | 1 |
| **Hospitalization for bleeding** | 4 (5.4) | 2 (3.0) | 0.7 |
| **STS score, mean (SD)** | 6.1 (4.3) | 5.8 (4.2) | 0.7 |
| **EuroSCORE I, mean (SD)** | 12.9 (10.5) | 12.7 (8.6) | 0.9 |
| **EuroSCORE II, mean (SD)** | 4.2 (2.8) | 5.1 (4.3) | 0.2 |
| **Edoxaban arm** | 74 (100) | 0 | **<0.0001** |
| **VKA arm** | 0 | 66 (100) | **<0.0001** |
| **Abnormal renal function** | 3 (4.1) | 1 (1.5) | 0.622 |
| **Intracranial hemorrhage** | 1 (1.4) | 1 (1.5) | 1 |
| **Abnormal liver function** | 0 | 0 |  |
| **Excessive alcohol use** | 0 | 2 (3.0) | 0.2 |
| **Hemoglobin, g/L, mean (SD)** | 108.4 (19.7) | 118.9 (143.4) | 0.5 |
| **Platelets, 10^9^/L, mean (SD)** | 173.9 (58.7) | 167.5 (57.6) | 0.5 |

Data presented as n (%) unless otherwise noted.

*Race was reported by the investigator from information obtained from patient history. “Other” includes patients of another race and those who chose not to report race. ^†^Cockcroft–Gault formula. ^‡^Persistent defined as irregular rhythm occurring between 8 and 364 days; long-standing persistent for >1 year. ^§^Criteria for adjustment of the edoxaban dose included CrCl ≤50 mL/min, bodyweight of ≤60 kg, and concomitant therapy with a P-glycoprotein inhibitor (both not used as dose adjustment criteria in US patients). **Chronic drug usage is one component of the HAS-BLED score, including antiplatelet agents, NSAIDs.

*AF* atrial fibrillation; *CABG* coronary artery bypass graft surgery; *CHA_2_DS_2_-VASc* congestive heart failure, hypertension, age ≥75 years, diabetes mellitus, stroke or TIA, vascular disease, age 65 to 74 years, sex category; *CNS* central nervous system; *COPD* chronic obstructive pulmonary disease; *CrCl* creatinine clearance; *HAS-BLED* hypertension, abnormal liver/renal function, stroke history, bleeding history or predisposition, labile INR, elderly, drug/alcohol usage; *INR* international normalized ratio; *NOAC* non–vitamin K antagonist oral anticoagulant; *NSAID* nonsteroidal anti-inflammatory drug; *OAP* oral antiplatelet; *PCI* percutaneous coronary intervention; *PPI* proton pump inhibitor; *SD* standard deviation; *STS* Society of Thoracic Surgeons; *TAVI* transcatheter aortic valve implantation; *TIA* transient ischemic attack; *VKA* vitamin K antagonist.

**Table S3** Site of bleeding for first major bleeding event in patients with a recent PCI history

| **Bleeding site** | **Edoxaban**  **(n = 73)** | **VKA**  **(n = 67)** | **Total**  **(N = 140)** |
| --- | --- | --- | --- |
| **Intracranial hemorrhage** | 1 | 3 | 4 |
| **Lower gastrointestinal** | 3 | 1 | 4 |
| **Upper gastrointestinal** | 4 | 4 | 8 |
| **Macroscopic hematuria/urethral** | 1 | 0 | 1 |
| **Puncture site** | 2 | 1 | 3 |
| **Surgical site** | 1 | 0 | 1 |
| **Other** | 2 | 1 | 3 |

Data shown are number of patients. Outcomes are reported for the on-treatment population (ie, patients on study medication or within 3 days of previous study medication).

*PCI* percutaneous coronary intervention; *VKA* vitamin K antagonist.

**Fig. S1** DAPT and SAPT use over time in patients with (a) and without (b) recent PCI history


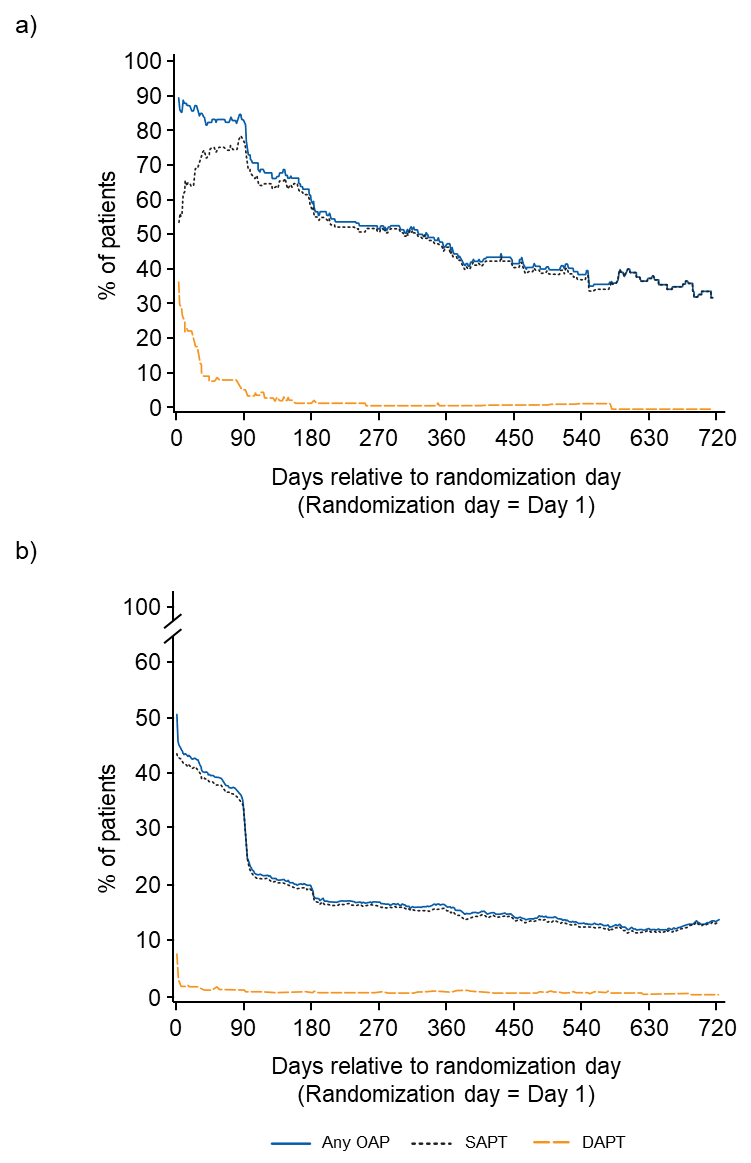


*DAPT* dual antiplatelet therapy; *OAP* oral antiplatelet therapy; *PCI* percutaneous coronary intervention; *SAPT* single antiplatelet therapy.

**Fig. S2** Time to NACE (a), major bleeding (b), and all-cause death (c) in patients with and without a recent PCI history

*
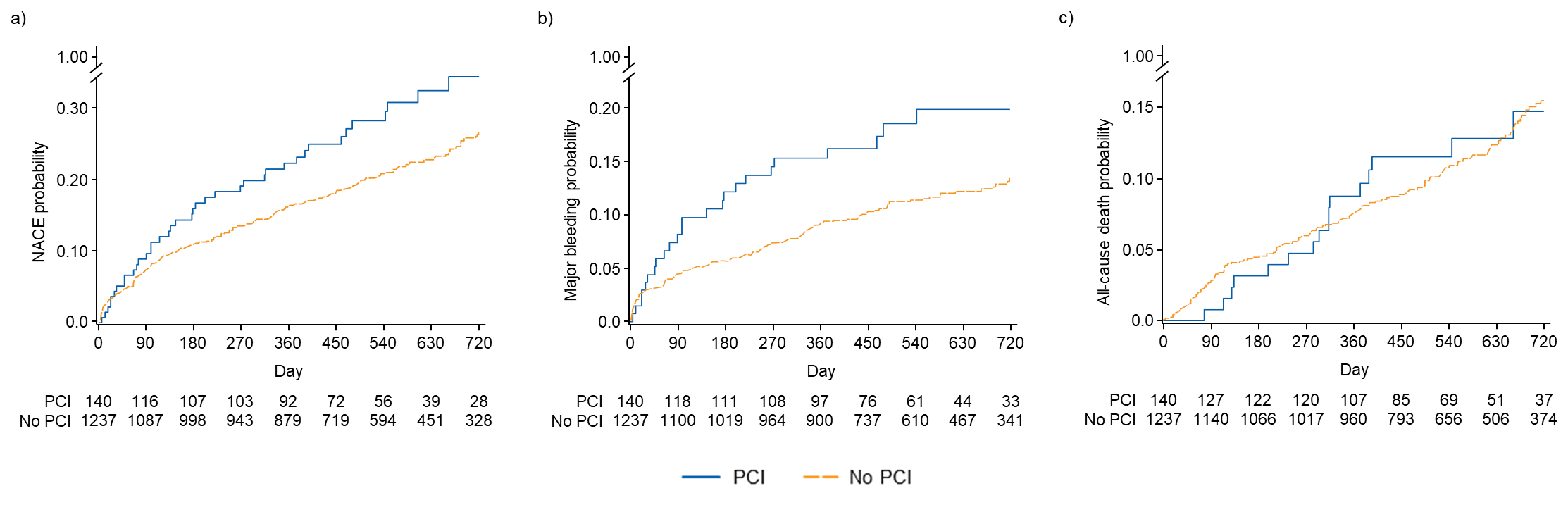
*

*NACE* net adverse clinical events; *PCI* percutaneous coronary intervention.
